# Genome-Wide Analysis of the First Sequenced *Mycoplasma capricolum* subsp. *capripneumoniae* Strain M1601

**DOI:** 10.1534/g3.117.300085

**Published:** 2017-07-27

**Authors:** Shengli Chen, Huafang Hao, Ping Zhao, François Thiaucourt, Ying He, Pengcheng Gao, Han Guo, Wenheng Ji, Zhanhui Wang, Zhongxin Lu, Yuefeng Chu, Yongsheng Liu

**Affiliations:** * State Key Laboratory of Veterinary Etiological Biology, Lanzhou Veterinary Research Institute, Chinese Academy of Agricultural Sciences, Lanzhou, 730046, Gansu, People’s Republic of China; † CIRAD, UMR CMAEE, F-34398 Montpellier, France; ‡ INRA, UMR1309 CMAEE, F-34398 Montpellier, France

**Keywords:** *Mycoplasma capricolum* subsp. *capripneumoniae*, genome, virulence factor, comparative analysis, phylogenetic analysis, Genome Report

## Abstract

*Mycoplasma capricolum* subsp. *capripneumoniae* (Mccp) is a common pathogen of goats that causes contagious caprine pleuropneumonia. We closed the gap and corrected rRNA operons in the draft genome of Mccp M1601: a strain isolated from an infected goat in a farm in Gansu, China. The genome size of M1601 is 1,016,707 bp with a GC content of 23.67%. We identified 915 genes (occupying 90.27% of the genome), of which 713 are protein-coding genes (excluding 163 pseudogenes). No genomic islands and complete insertion sequences were found in the genome. Putative determinants associated with the organism’s virulence were analyzed, and 26 genes (including one adhesion protein gene, two capsule synthesis gene clusters, two lipoproteins, hemolysin A, ClpB, and proteins involved in pyruvate metabolism and cation transport) were potential virulence factors. In addition, two transporter systems (ATP-binding cassette [ABC] transporters and phosphotransferase) and two secretion systems (Sec and signal recognition particle [SRP] pathways) were observed in the Mccp genome. Genome synteny analysis reveals a good collinear relationship between M1601 and Mccp type strain F38. Phylogenetic analysis based on 11 single-copy core genes of 31 *Mycoplasma* strains revealed good collinearity between M1601 and *Mycoplasma capricolum* subsp. *capricolum* (Mcc) and close relationship among *Mycoplasma mycoides* cluster strains. Our genome-wide analysis of Mccp M1601 provides helpful information on the pathogenic mechanisms and genetics of Mccp.

*Mycoplasma capricolum* subsp. *capripneumoniae* (Mccp) is the causative agent for contagious caprine pleuropneumonia (CCPP), a major infectious disease characterized by high morbidity in goats and its ability to cause considerable economic losses in Africa and Asia. The pathological lesions of CCPP are localized exclusively in the lungs and pleura, and the pathological changes consist of a pleuropneumonia, unilateral hepatization, pleuritis, and an accumulation of pleural fluid (OIE 2016). The disease is now threatening disease-free countries and has been listed by the World Organisation for Animal Health (OIE) (Nicholas and Churchward 2012).

Mccp belongs to the genus *Mycoplasma* under the class *Mollicutes*. Isolating Mccp requires a high level of expertise and special growth medium. In addition, the organism is very fastidious and has slow growth. Until now, only about half of countries, where clinical disease has been reported, had isolated the causative organism (Manso-Silván *et al.* 2011).

In 2007, a severe contagious respiratory disease occurred in a goat farm in the Gansu Province of China that has spread to other provinces. The main symptoms of this disease were coughing and high fever, which led to a morbidity rate of 62% and a mortality rate of 45%. The clinical symptoms and pathological changes were similar to CCPP. The organism was isolated from lungs of sick goats and placed in improved Thiaucourt’s medium, purified three times, and named M1601. Biochemistry tests, 16S RNA sequence analysis, and animal pathogenicity tests were performed to further confirm the Mccp strain (Guo *et al.* 2011).

In 2011, the first draft genome of Mccp strain M1601 was released (Chu *et al.* 2011). The genome of four other Mccp strains was reported later (Dupuy and Thiaucourt 2014; Falquet *et al.* 2014), but virulence factors of this important pathogen are still poorly understood. In this study, the gap of M1601 draft genome was closed, and the rRNA operon sequences were corrected to yield complete genomic sequences. Comprehensive genomic analysis of this pathogen was conducted. Putative determinants associated with Mccp virulence were identified based on the comprehensive genome analysis. Finally, comparative and phylogenetic analyses were performed. Understanding the supposed virulence genes, genome features, and genetics of this strain would be valuable in determining its pathogenic mechanisms and genetics.

## Materials and Methods

### Bacterial growth and DNA extraction

Mccp M1601 was grown in improved Thiaucourt’s medium (PPLO broth 21 g/liter, glucose 2 g/liter, sodium pyruvate 2 g/liter, 20% horse serum, 25% yeast extract 100 ml/liter, 0.4% phenol red 5 ml/liter, 100 units of penicillin, and 0.01% acetic acid thallium) at 37°. The 100 ml mid-log phase culture was harvested by centrifugation at 12,000 × *g* for 30 min and resuspended in 10 ml PBS (0.01 M, pH 7.2). Subsequently, total genomic DNA was extracted with a TIANamp Bacteria DNA Kit (Tiangen, Beijing, China) according to manufacturer’s instructions.

### Gap closure and rRNA operon sequences correction

The M1601 draft genome sequence with one gap cited in 760,982–761,498 was previously described (Chu *et al.* 2011). Gap closure was conducted as follows: The corresponding sequence was extracted from Mccp Abomsa-9231 sequence (NZ_LM995445) and used as a reference template for assembly. The assembly was then corrected manually. The gap consisted of 517 bp, and it was then inserted into the M1601 draft genome. There are two sets of rRNA operon sequence in the genome, one operon of M1601 strain was PCR amplified and sequenced by Sanger method. Another operon sequence was deducted after performing an assembly with the previous operon, being chosen as the reference sequence. The corrected rRNA operon sequences were then replaced in the corresponding M1601 sequence, yielding complete genomic sequences (GenBank under accession number NZ_CP017125).

### Annotation and sequence analysis

The complete sequence was analyzed using Glimmer 3.0 (Delcher *et al.* 1999) for open reading frames containing >30 predicted amino acid residues. Transfer RNA (tRNA) and ribosomal RNA (rRNA) genes were predicted using tRNAscan-SE (Lowe and Eddy 1997) and Aragorn (Laslett and Canback 2004), and RNAmmer (Lagesen *et al.* 2007), respectively. Insertion and deletion (InDel) detection was conducted using LASTZ software (Harris 2007) to compare M1601 with *Mycoplasma capricolum* subsp. *capricolum* (Mcc) reference strain 27343. The best match results (<10 bp) were then extracted by using axtBest to obtain the preliminary InDel results. The 150 bp (3 × SD) from upstream and downstream of the reference sequence InDel sites were aligned and validated with the sample sequencing reads by BWA software (Li and Durbin 2009). After filtering, the reliable InDel sites were obtained. The genomic islands and insertion sequences were found by using Path-DIOMB (Hsiao *et al.* 2003) and ISfinder (https://www-is.biotoul.fr/), respectively.

The function annotation of the predicted protein-coding genes was conducted by blasting based on the COG, KEGG, Swiss-Prot, TrEMBL, and NCBI-NR databases. Pseudogenes were detected by BLASTN analysis, and then the annotation was revised manually.

The putative virulence genes were identified by gene annotation and reference studies (O’Riordan *et al.* 2003; Chastanet *et al.* 2004; Hames *et al.* 2009; Bürki *et al.* 2015; Gründel *et al.* 2015). BLASTP searches (*E*-value <1e−5) against the NCBI database were applied, and the results were filtered by selecting the highest score of alignment (homology identity >40% and minimal alignment length percentage >40%). Core genes and specific genes were analyzed by CD-HIT software (Li and Godzik 2006) for clustering similar proteins with a threshold of 50% pairwise identity and 0.7 length difference cutoff in amino acids.

### Comparative and phylogenetic analysis

Genomic alignment of Mccp strains M1601 and F38 was conducted using MUMmer (Delcher *et al.* 2003) and LASTZ (Harris 2007). Genomic synteny was performed based on the alignment results. Multiple sequence alignments of single-copy of core genes among 31 *Mycoplasma* strains were performed using MUSCLE (Edgar 2004). The phylogenetic tree was constructed by TreeBeST (Nandi *et al.* 2010) using the maximum likelihood method with 1000 bootstrap replicates. The genome sequences of other *Mycoplasma* strains were downloaded from the NCBI database.

### Data availability

The genome sequence data were deposited in GenBank with the accession number NZ_CP017125. Supplemental Material, Figure S1 shows a comparison of genomic structure between Mccp strain M1601 and Mcc ATCC 27343. Figure S2 shows a comparison of complete genome between Mccp strains M1601 and F38. Table S1 shows an overview of the predicted results of the Mccp M1601 genome. Table S2 shows the genes involved in transport and metabolism. Table S3 shows the transporter system of Mccp. Table S4 shows the proteins involved in the secretion system. Table S5 shows the predicted genes involved in DNA replication. Table S6 shows the predicted genes involved in transcription. Table S7 shows the predicted genes involved in translation. Table S8 shows the InDel analysis between the M1601 genome and reference strain sequence. Table S9 shows the genome information of Mccp strain M1601 and four other partially annotated Mccp strains.

## Results

### Genome features

**Figure 1:**
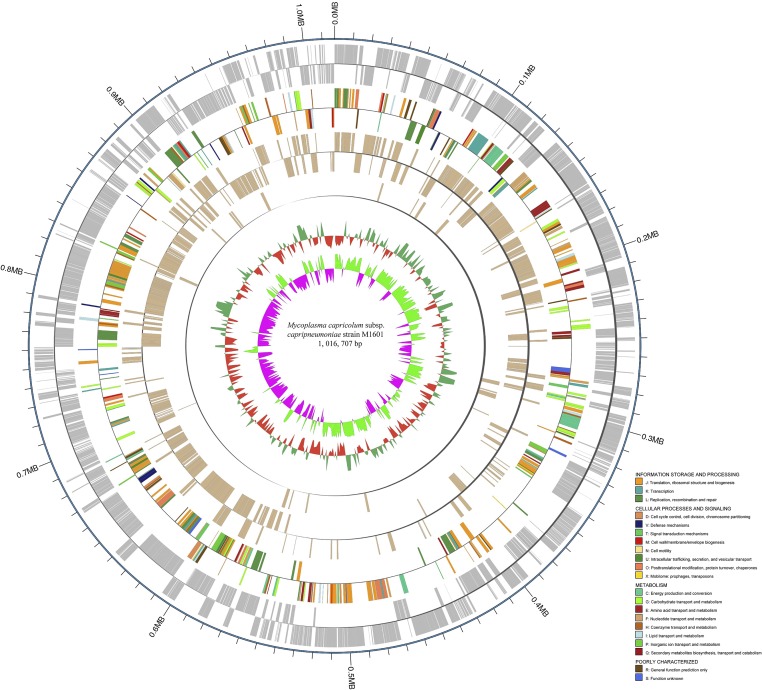
Genome architecture of Mccp strain M1601. The *dnaA* gene is at position 1. Moving inside, the first circle shows the position coordinates of genome sequence. The second circle represents the locations of the predicted coding sequences on the plus and minus strands. The third circle shows the results of color-coded CDS by COG categories annotation (see the description in the bottom right corner). The fourth circle shows the presence of orthologous genes in five Mccp strains and Mcc reference strain 27343. The fifth circle represents the location of supposed virulence genes. The sixth circle represents the mean centered G + C content of the genome. The average GC is baseline, outwardly projecting expresses higher than the average, and inwardly projecting means below. The seventh circle shows the GC (G + C) skew plot. Green: above zero; purple: below zero.

The Mccp strain M1601 genome contains a single, circular chromosome of 1,016,707 bp with GC content of 23.67%, in line with the low GC content characteristics of *Mycoplasma* (Figure 1). A total of 915 genes were identified and occupy 90.27% of the genome. The genome contains 713 protein-coding genes (excluding 163 pseudogenes), six rRNA genes, 30 tRNA genes, and three ncRNA genes (Table S1). Among the protein-coding genes, 461 genes (50.38%) were assigned into specific functional clusters of orthologous groups families, comprising 21 functional categories (Table 1). No genomic islands and complete insertion sequences were detected in the genome. The genome sequence data were deposited in GenBank with the accession number NZ_CP017125.

**Table 1:** Functional category in COG of Mccp

Code	Functional category	F38	M1601	Common
C	Energy production and conversion	25	25	25
D	Cell cycle control, cell division, chromosome partitioning	5	5	5
E	Amino acid transport and metabolism	32	30	30
F	Nucleotide transport and metabolism	29	29	29
G	Carbohydrate transport and metabolism	42	41	41
H	Coenzyme transport and metabolism	22	20	20
I	Lipid transport and metabolism	13	13	13
J	Translation, ribosomal structure and biogenesis	139	138	138
K	Transcription	26	26	26
L	Replication, recombination and repair	43	44	43
M	Cell wall/membrane/envelope biogenesis	8	8	8
N	Cell motility	2	2	2
O	Posttranslational modification, protein turnover, chaperones	18	18	18
P	Inorganic ion transport and metabolism	21	22	21
Q	Secondary metabolites biosynthesis, transport and catabolism	2	2	2
R	General function prediction only	34	32	32
S	Function unknown	8	9	8
T	Signal transduction mechanisms	13	13	13
U	Intracellular trafficking, secretion, and vesicular transport	5	5	5
V	Defense mechanisms	10	10	10
X	Mobilome: prophages, transposons	3	3	3
—	Total in COG	465	461	458

### Virulence factors

Adhesion is the first step of *Mycoplasma* infection of host cells; thus, adhesion proteins can be regarded as virulence-associated proteins of the pathogen (Razin *et al.* 1998). One adhesion-related gene (XDU01000267) was found in the Mccp M1601 genome. The capsule is often thought to be an important virulence factor for some pathogenic bacterium, such as *Pasteurella multocida* (Boyce and Adler 2000) and *Mycoplasma mycoides* subsp. *mycoides SC* (March and Brodlie 2000; Pilo *et al.* 2007). The genome contains a gene cluster (XDU01000075, XDU01000076, XDU01000814, and XDU01000816) involved in the synthesis of the capsule, comprising genes encoding glycosyltransferase, UTP–glucose-1-phosphate uridylyltransferase, and diacylglyceryl transferase (Table 2).

**Table 2:** Potential virulence factors involved in the M1601 genome

Locus	Product	Gene	Gene length (bp)	Site	Prevalence in other four Mccp genome*^a^*	Prevalence in mycoplasma strain*^b^*
XDU01000037	P60 surface lipoprotein	*p60*	1644	46190..47833	100% (4/4)	30.8% (8/26)
XDU01000067	Hemolysin A	—	729	84200..84928	100% (4/4)	57.7% (15/26)
XDU01000075	Glycosyl transferase	—	1575	90225..91799	100% (4/4)	84.6% (22/26)
XDU01000076	UTP–glucose-1-phosphate uridylyltransferase	*galU*	879	91802..92680	100% (4/4)	57.7% (15/26)
XDU01000099	Magnesium-translocating P-type ATPase	*mgtA*	2829	117833..120661	100% (4/4)	50.0% (13/26)
XDU01000242	Glycerol facilitator factor	*glpF*	780	288715..289494	100% (4/4)	50.0% (13/26)
XDU01000243	Glycerol kinase	*glpK*	1149	289530..290678	100% (4/4)	73.1% (19/26)
XDU01000244	Glycerol-3-phosphate dehydrogenase	*glpD*	1164	291065..292228	100% (4/4)	88.5% (23/26)
XDU01000249	Lipoate-protein ligase A	*lplA*	1005	299248..300252	100% (4/4)	65.4% (17/26)
XDU01000251	Pyruvate dehydrogenase E1 component subunit beta	*pdhB*	990	301399..302388	100% (4/4)	80.8% (21/26)
XDU01000252	Branched-chain alpha-keto acid dehydrogenase subunit E2	*pdhC*	1317	302417..303733	100% (4/4)	50.0% (13/26)
XDU01000253	Dihydrolipoamide dehydrogenase (E3) component	*pdhD*	1890	303753..305642	100% (4/4)	84.6% (22/26)
XDU01000267	Adhesion-related protein	—	717	321984..322700	100% (4/4)	26.9% (7/26)
XDU01000405	Chaperone protein ClpB	*clpB*	2142	474287..476428	100% (4/4)	76.9% (20/26)
XDU01000466	Dihydrolipoamide dehydrogenase (E3) component	*pdhD*	1362	535434..536795	100% (4/4)	57.7% (15/26)
XDU01000483	Lipoate-protein ligase A	*lplA*	1038	552430..553467	100% (4/4)	46.2% (12/26)
XDU01000486	ABC transporter permease	*gtsC*	804	557034..557837	100% (4/4)	46.2% (12/26)
XDU01000487	Glycerol ABC transporter permease	*gtsB*	1005	557815..558819	100% (4/4)	46.2% (12/26)
XDU01000488	ABC transporter ATP-binding protein	*gtsA*	1212	558812..560023	100% (4/4)	53.8% (14/26)
XDU01000612	Lipoproteins VmcC	—	399	699233..699631	100% (4/4)	23.1% (6/26)
XDU01000742	Sodium transporter	—	1602	828031..829632	100% (4/4)	30.8% (8/26)
XDU01000743	Potassium transporter TrkA	*trkA*	729	829711..830439	100% (4/4)	30.8% (8/26)
XDU01000796	Magnesium transporter	*mgtE*	1404	884553..885956	100% (4/4)	73.1% (19/26)
XDU01000814	Diacylglyceryl transferase	—	1581	907200..908780	100% (4/4)	57.7% (15/26)
XDU01000816	Diacylglyceryl transferase	—	1431	909705..911135	100% (4/4)	69.2% (18/26)
XDU01000848	Magnesium transporter	*mgtA*	1116	944515..945630	100% (4/4)	30.8% (8/26)

a Mccp strains including F38 (NZ_LN515398), ILRI181(NZ_LN515399), 87001(NZ_CP006959), and 9231(NZ_LM995445).

b Twenty-six other species of *Mycoplasma* strains for which genome is used for the phylogenetic analysis except the five Mccp strains.

ClpC is an ATPase that plays an important role in cell adhesion and invasion and is responsible for the virulence of *L. monocytogenes* (Nair *et al.* 2000). ClpB is a component of stress response in microorganisms that serve as a chaperone for preventing protein aggregation and assisting in the refolding of denatured proteins. ClpB was also involved in the virulence of *L. monocytogenes* (Chastanet *et al.* 2004). Although *clpC* gene was not found, one *clpB* gene (XDU01000405) was identified in the genome of Mccp, and it shows 72% identity with the ClpB protein of *L. monocytogenes*. Thus, ClpB may be a virulence factor of Mccp.

Variable surface proteins (Vsps) have been thought to play an important role in the process of antigenic variation and immunity evasion, and are regarded to be a pathogenic factor for *Mycoplasma* (Bürki *et al.* 2015). VmcC is reported to play key role in the antigenic variation and survival of Mcc (Wise *et al.* 2006), and P60 surface lipoprotein is considered to be related to virulence of *M. hyopneumoniae* (Seymour *et al.* 2012). One VmcC lipoprotein (XDU01000612) and P60 surface lipoprotein (XDU01000037) were found in the genome. These identified lipoproteins may be associated with Mccp virulence.

Hemolysins are toxic proteins that cause the lysis of erythrocytes by forming pores in their membranes (Goebel *et al.* 1988). Hemolysin A (XDU01000067) was identified in the Mccp genome, and it could be considered as a virulence factor.

Pyruvate is the first product in the process of aerobic metabolism of glucose. It goes to the mitochondrion to produce acetyl-CoA under catalysis of pyruvate dehydrogenase (PDH) enzyme complex. Lipoate–protein ligase (LplA) and PDH complex (composed of PDH E1, lipoic acid acetyltransferase E2, and dihydrolipoamide dehydrogenase E3) play a critical role in pyruvate metabolism (Patel *et al.* 2014). A mutant of dihydrolipoamide dehydrogenase E3 was significantly attenuated in *M. gallisepticum* *in vivo* (Gates *et al.* 2008). Recent research indicated that pyruvate metabolism component PDH subunits may contribute to the pathogenesis of *M. pneumoniae* infections by interaction with human plasminogen (Gründel *et al.* 2015). LplA ligates lipoic acid from the host to the PDH E2 component to generate E2-lipoamide, which plays an important role in pyruvate metabolism. *L. monocytogenes* lacking LplA1 were defective for growth in the host cytosol and attenuated 300-fold compared with wild-type strain (O’Riordan *et al.* 2003). Four PDH complex genes and two *lplA* genes were identified in the genome, and they were regarded to be virulence factors (Table 2).

Glycerol metabolism and production of H_2_O_2_ are considered to be associated with *Mycoplasma* virulence (Vilei and Frey 2001; Hames *et al.* 2009). The *glpF-glpK-glpD* (XDU01000242, XDU01000243, and XDU01000244) and *gtsA-gtsB-gtsC* gene clusters (XDU01000486, XDU01000487, and XDU01000488), which are involved in glycerol metabolism, were identified in the Mccp genome.

Magnesium transporters MgtA and MgtE have been showed to be related to virulence of some bacteria (Groisman *et al.* 2013), such as *Aeromonas hydrophila* (Merino *et al.* 2001). In the Mccp genome, three magnesium transporters genes (XDU01000099, XDU01000796, XDU01000848) were found. Potassium transporter TrkA is related to virulence of *Salmonella* (Su *et al.* 2009), and sodium transporter is reported to be associated with the virulence of *Yersinia pestis* (Minato *et al.* 2013) and *Pseudomonas aeruginosa* (Ueda and Wood 2008). One potassium transporter TrkA (XDU01000743) and one sodium transporter (XDU01000742) were found in the genome, which are involved in potassium and sodium uptake, respectively. These proteins may be associated with the virulence of Mccp.

### Transporter, metabolism, and secretion

The biosynthetic capacity of *Mycoplasma* is severely poor, and most nutrition is obtained from the host during the intracellular lifestyle (Roger and Robin 1998). Thus, many genes are involved in *Mycoplasma* transporter and metabolism systems. In the M1601 genome, 175 genes were identified, which were related to transporter and metabolism systems (Table S2). In total, 164 genes were involved in amino acid, nucleotide, carbohydrate, inorganic ion, and coenzyme transport and metabolism, whereas 11 other genes were related with lipid and secondary metabolite biosynthesis, transport, and catabolism. Two transporter systems, ABC transporter system and the phosphotransferase system (PTS), were identified. Forty-eight genes encode the ABC-type transporter systems including 22 ABC transporter ATP-binding proteins, 22 ABC transporter permeases, two ATPase components, and two other proteins. By contrast, 18 genes encode for the PTS transport system (Table S3).

Protein secretion systems are also important for *Mycoplasma* survival in the host. The Mccp encodes nine proteins that are involved in protein secretion systems, including the Sec and SRP pathways (Table S4). The Sec system contains six proteins, SecA, SecD, SecE, SecG, SecY, and YidC. SRP-docking proteins FtsY and ffh were identified to participate in the SRP pathway. In addition, one lipoprotein signal peptidase A8 (XDU01000432) was found, whereas the signal peptidase I gene was absent.

### Replication, transcription, and translation

In the Mccp genome, *dnaA* encoded by XDU01000001 binds to the DnaA box as an ATP-bound complex at the origin of replication, during the initiation of chromosomal replication. Fifty genes, which encode DNA polymerase III, DNA helicase, DNA polymerase I, 5′-3′ exonuclease, endonuclease, repair protein, and NAD-dependent DNA ligase, were found to be involved in replication, recombination, and repair (Table S5).

In total, 27 genes (Table S6) were involved in transcription whereas 108 genes (Table S7) were related to translation, ribosomal structure, and biogenesis. Transcription elongation and termination were regulated by NusA, NusB, NusG, and GreA. GreA is a transcription elongation factor which could prevent transcription arrest, and NusA can induce transcription pausing, or stimulate anti-termination together with NusB and NusG (Borukhov *et al.* 2005). Eight transcriptional regulators were found in the Mccp genome, which include two RpiR and DeoR, one ROK, GntR, HrcA, and Fur (Table S6). In addition, 50 ribosomal proteins, 21 tRNA synthetase genes, and 11 translation factors were found in the genome (Table S7).

### Comparative and evolutionary analysis

To reveal the genetic relationship between Mccp and Mcc, the Mccp strain M1601 genome and Mcc reference strain ATCC27343 (NC_007633) were compared (Figure S1). The size of the Mcc ATCC 27343 genome was 6684 bp shorter than the Mccp M1601 genome. For 870 genes in the Mcc ATCC 27343, 79.43% of them are observed in the Mccp genome. No large-scale insertion, deletions, and inversions existed between these two genomes, whilst there were 416 insertion/deletions found, these included 196 (47.12%) insertions and 220 (52.88%) deletions (Table S8). The genome comparisons among the sequenced Mccp strain M1601 and four other annotated Mccp strains were also conducted and are listed in Table S9.

In addition, the synteny of M1601 and the reference genome Mccp F38 was analyzed. Three blocks were developed with 99.86% identity. The block type exhibited forward collinearity, non-translocation, and non-inversion. Relative to the F38 genome, 870 bp deficiency and 446 bp insertion existed between the first two blocks and the last two blocks in M1601. Therefore, a good collinear relationship exists between M1601 and F38 (Figure S2).

**Figure 2:**
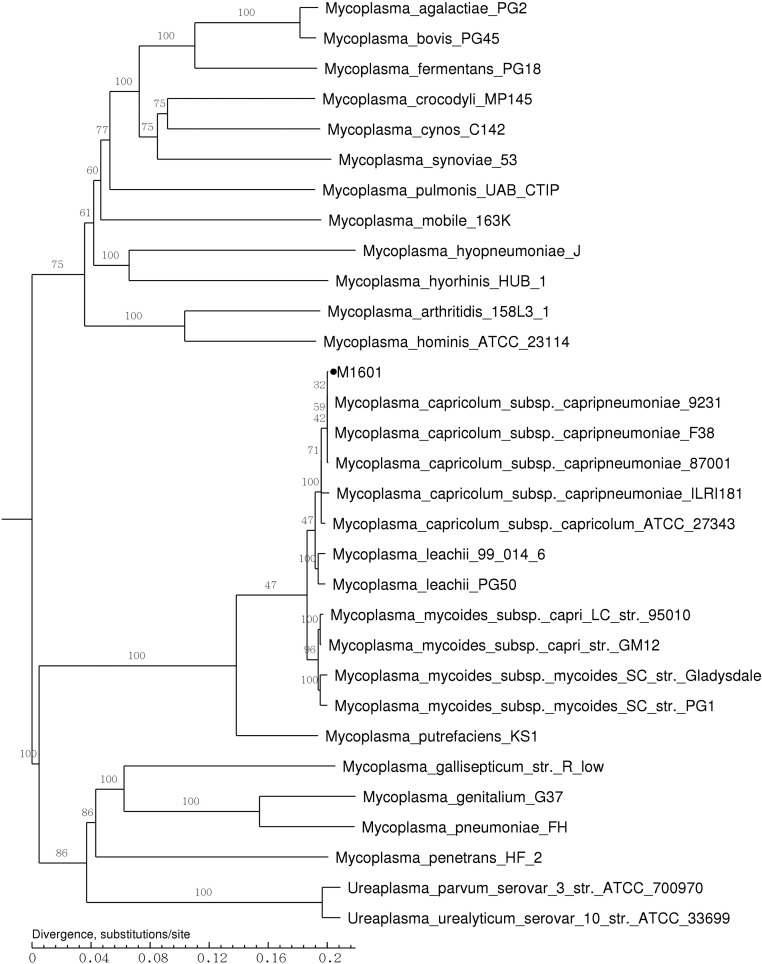
Phylogenetic tree based on 11 single-copy core genes of 31 selected *Mycoplasma*. The phylogenetic tree was constructed by TreeBeST using the maximum likelihood method with 1000 bootstrap replicates. The bootstrap numbers are given for each node. The tree is drawn to scale, with branch lengths measured in the number of substitutions per site. The Mccp strain M1601 is highlighted by black circles.

A phylogenetic tree based on 11 single-copy core genes of 31 *Mycoplasma* strains was constructed (Figure 2). The results indicated a close relationship between Mccp M1601 and four other strains. All strains of Mccp, *Mycoplasma mycoides* subsp. *mycoides SC* (MmmSC), *M. mycoides* subsp. *capri* (Mmc), Mcc, and *M. leachii* (Ml), belong to a large *M. mycoides* cluster.

## Discussion

Adhering to host cell is a crucial step in the process of *Mycoplasma* infection and colonization, and is an important aspect of the research of pathogenic mechanisms. At present, several adhesion proteins of various *Mycoplasma* species have been identified, such as variable surface lipoproteins (Sachse *et al.* 2000), α-enolase (Song *et al.* 2012), VpmaX protein (Zou *et al.* 2013) of *M. bovis*, P50 of *M. hominis* (Kitzerow *et al.* 1999), LppS of *M. conjunctivae* (Belloy *et al.* 2013), and P19 of *M. mycoides* subsp. *mycoides* (Mmm) (Zhou *et al.* 2016). A previous study reported that a hypothetical membrane protein encoded by the 0297 gene of Mccp strain C87001 showed significant adhesion on goat bronchial epithelial cells (Bai *et al.* 2014). The XDU01000267 gene, corresponding to homologous 0297 gene, may be an adhesion protein of Mccp, but this finding needs further verification.

Vsps play an important role in *Mycoplasma* colonization and adaptation to the host environment in different infection stages. Vsps are also related to antigenicity and immune regulation of *Mycoplasma* (Buchenau *et al.* 2010; Bolland and Dybvig 2012). Variable surface lipoprotein gene cluster exists in many *Mycoplasma* genomes, such as Mmc, Mcc, and MmmSC. In M1601, one VmcC and one P60 surface lipoprotein were found, and both were considered to be potential virulence factors. In addition, proteins related to capsule synthesis and pyruvate metabolism were also related with bacterial virulence (Boyce and Adler 2000; O’Riordan *et al.* 2003; Gates *et al.* 2008; Gründel *et al.* 2015). We found two gene clusters involved in capsule synthesis and six pyruvate-metabolism-related enzyme genes in the Mccp genome, and all these genes may be associated with Mccp virulence.

At present, seven types of protein secretion systems in bacteria have been identified (Abdallah *et al.* 2007). A signal peptide present at N-terminal on the secreted protein via Sec pathway is required and cleaved to the mature form (Beckwith 2013). Signal peptidases are proteases that remove the N-terminal signal peptide of secreted proteins in the endoplasmic reticulum. Signal peptidase I gene and lipoprotein signal peptidase gene are found in *Mycoplasma* species such as *M. conjunctivae* (Calderon-Copete *et al.* 2009), *M. hyopneumoniae* (Moitinho-Silva *et al.* 2012), *M. pulmonis* (Chambaud *et al.* 2001), and *M. synoviae* (Vasconcelos *et al.* 2005). However, in the Mccp M1601 genome, only one lipoprotein signal peptidase gene was found, which was similar to *M. bovis* (Li *et al.* 2011). This finding indicated that Mccp may have the same mechanism of extracellular protein secretion as *M. bovis* but different from *M. pulmonis*, *M. hyopneumoniae*, and *M. synoviae*.

DNA replication, recombination, and repair in the Mccp genome were also analyzed, and 50 proteins were involved in these biological processes. However, no typical mismatch-repair system (MutHLS) genes were found. The error may be repaired mainly by RecF pathway which including recombinational repair, the nucleotide excision repair system and the base excision repair system as previously reported (Carvalho *et al.* 2005).

M1601 and Mcc strain ATCC 27343 kept good collinearity, suggesting closer genetic relationship between Mccp and Mcc. Good collinear relationship was seen for Mccp strains M1601 and F38. The phylogenetic analysis indicated that Mccp, Mmc, Mcc, Mmm, and Ml all belong to the Mm cluster, which is in accordance with the result of collinearity analysis and morphological and biochemical features.

## Supplementary Material

Supplemental material is available online at www.g3journal.org/lookup/suppl/doi:10.1534/g3.117.300085/-/DC1.
