# Supplementary material for: Genome-Wide Analysis of the First Sequenced Mycoplasma capricolum subsp. capripneumoniae Strain M1601
Source: G3 (Bethesda). 2017 Jul 27;7(9):2899–906. doi: 10.1534/g3.117.300085 (PMC5592918; doi:10.1534/g3.117.300085)
Supplement: Supplementary file 1 [file 2899FigureS1.doc]

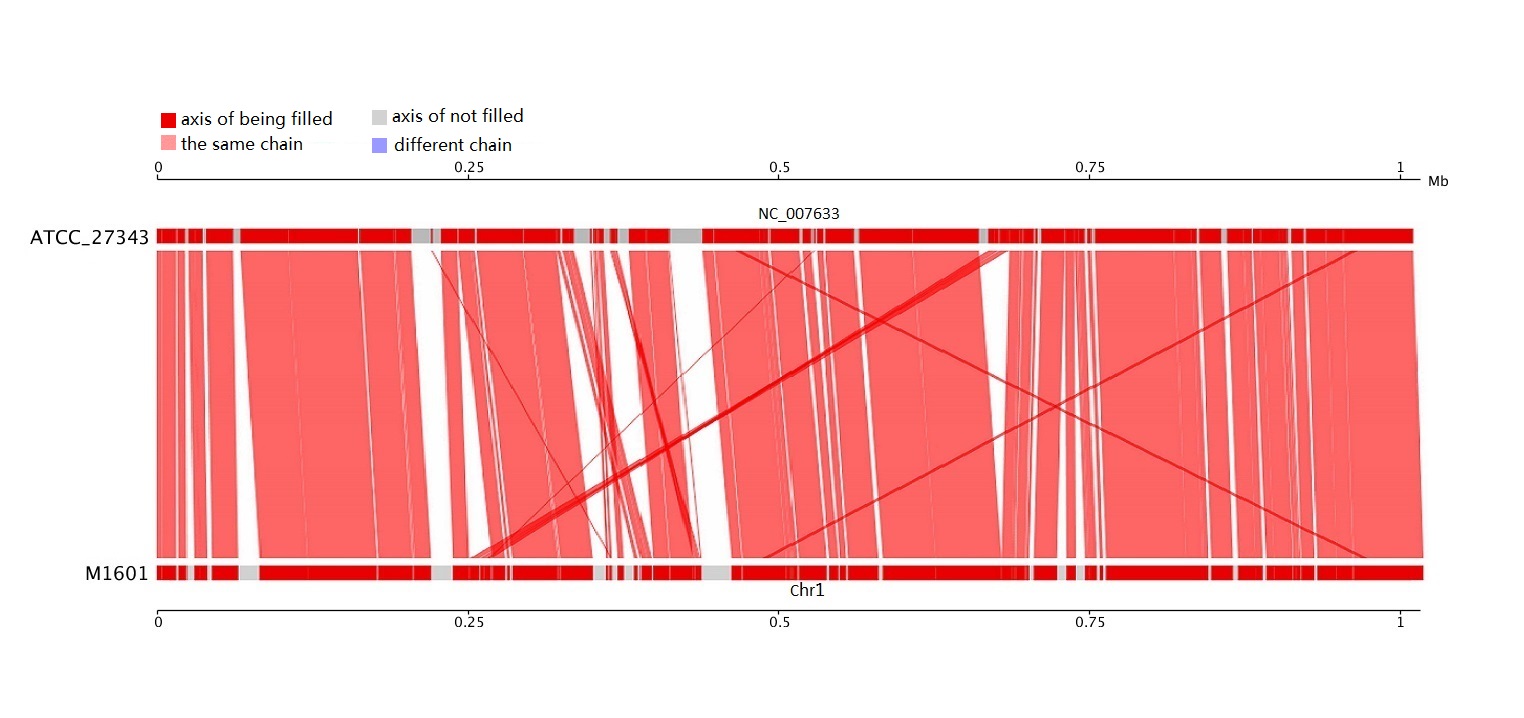


**Figure S1.** Comparison of genomic structure between Mccp strain M1601 and Mcc ATCC 27343. The upper axis is the reference strain genome and the lower is M1601 genome. Red on the axis represents homologous sequences between the two genomes and gray represents no homologous sequences existed. Red line between the upper and lower axises represents homologous sequences with the same direction between the two genomes.
