# Supplementary material for: Genome-Wide Analysis of the First Sequenced Mycoplasma capricolum subsp. capripneumoniae Strain M1601
Source: G3 (Bethesda). 2017 Jul 27;7(9):2899–906. doi: 10.1534/g3.117.300085 (PMC5592918; doi:10.1534/g3.117.300085)
Supplement: Supplementary file 2 [file 2899FigureS2.doc]

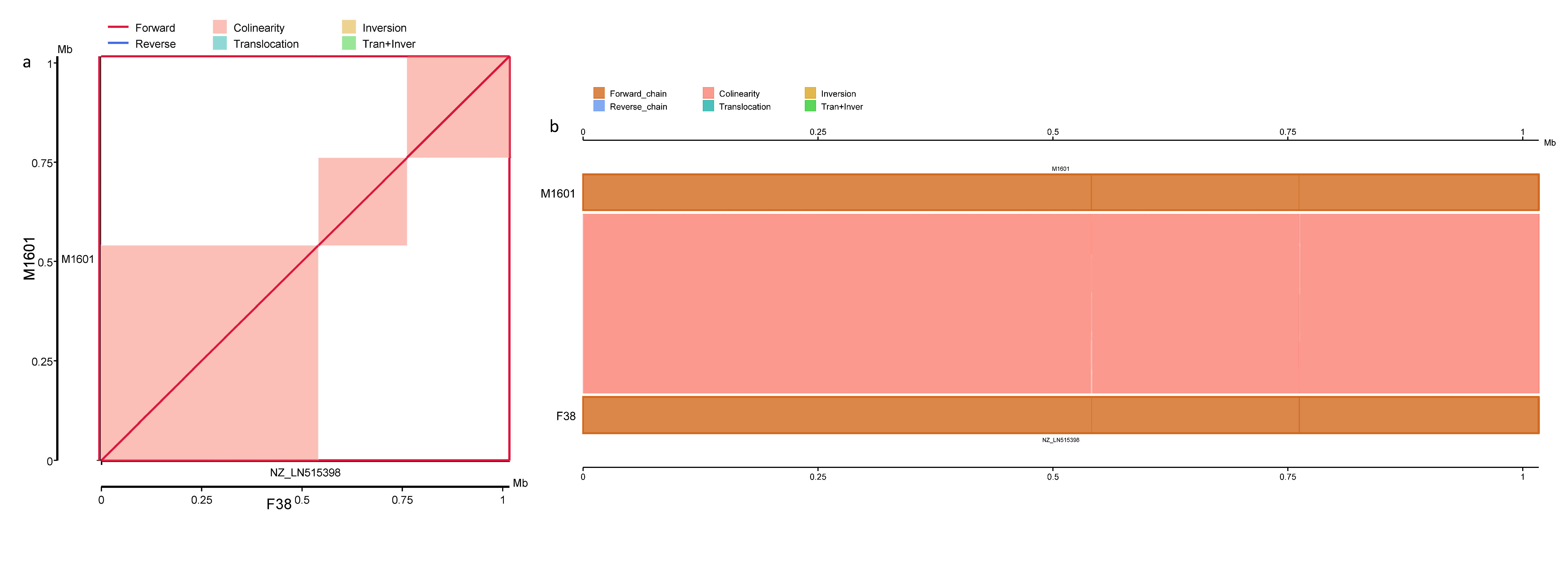


**Figure S2.** Comparison of complete genome between Mccp strains M1601 and F38. a: The display of two genomes on two-dimensional collinear. The vertical and horizontal axises represent the sequenced genome and referenced genome, respectively. The red line means forward alignment and pink module indicates collinear between them. b: The display of two whole genomes on parallel collinear. The upper shaft is the reference strain genome and the lower is sequenced genome. The yellow box in the upper and lower axises indicate the genomic positive strand. The filled color in the box denotes alignment similarity and the complete filling indicates 100% similarity.
