# Supplementary material for: Genome-Wide Analysis of the First Sequenced Mycoplasma capricolum subsp. capripneumoniae Strain M1601
Source: G3 (Bethesda). 2017 Jul 27;7(9):2899–906. doi: 10.1534/g3.117.300085 (PMC5592918; doi:10.1534/g3.117.300085)
Supplement: Supplementary file 3 [file 2899TableS1.doc]

**Table S1 Overview of the predicted results of Mccp M1601 genome**

| Description | Results |
| --- | --- |
| Genome size(bp) | 1,016,707 |
| Gene number | 915 |
| Protein-coding genes (excluding pseudogenes) | 713 |
| Gene length (bp) | 917,822 |
| GC content | 23.67% |
| GC content in gene region | 24.21% |
| Gene/Genome (%) | 90.27% |
| Gene average length (bp) | 1003 |
| Intergenic region length (bp) | 98,885 |
| GC content in intergenic region | 18.66% |
| Intergenic length/Genome (%) | 9.73% |
| Pseudogenes | 163 |
| tRNA number | 30 |
| tRNA average length | 78.36 |
| rRNA (by de novo prediction) | 6 |
| rRNA (by homology prediction) | 6 |
| tRNA average length | 1509 |
| ncRNA | 3 |
