# Supplementary material for: Genome-Wide Analysis of the First Sequenced Mycoplasma capricolum subsp. capripneumoniae Strain M1601
Source: G3 (Bethesda). 2017 Jul 27;7(9):2899–906. doi: 10.1534/g3.117.300085 (PMC5592918; doi:10.1534/g3.117.300085)
Supplement: Supplementary file 4 [file 2899TableS2.doc]

**Table S2 The genes involved in transport and metabolism**

| Locus | Product | Gene | Gene length (bp) | Protein length (aa) |
| --- | --- | --- | --- | --- |
| 3 | Secondary metabolites biosynthesis, transport and catabolism |  |  |  |
| XDU01000029 | sorbitol 6-phosphate dehydrogenase | - | 759 | 252 |
| XDU01000683 | acyl carrier protein | *acpP* | 222 | 73 |
| XDU01000898 | isochorismatase | - | 501 | 166 |
| 8 | Lipid transport and metabolism |  |  |  |
| XDU01000022 | FMN-dependent NADH-azoreductase | *azoR* | 600 | 199 |
| XDU01000029 | sorbitol 6-phosphate dehydrogenase | - | 759 | 252 |
| XDU01000160 | cardiolipin synthase | *cls* | 1530 | 509 |
| XDU01000370 | phosphatidate cytidylyltransferase | *cdsA* | 1029 | 342 |
| XDU01000525 | phosphate acyltransferase | *plsX* | 1005 | 334 |
| XDU01000683 | acyl carrier protein | *acpP* | 222 | 73 |
| XDU01000844 | CDP-diacylglycerol--glycerol-3-phosphate 3-phosphatidyltransferase | *pgsA* | 597 | 198 |
| XDU01000886 | lysophospholipase | - | 906 | 301 |
| 15 | Coenzyme transport and metabolism |  |  |  |
| XDU01000066 | dephospho-CoA kinase | - | 555 | 184 |
| XDU01000073 | riboflavin kinase | - | 753 | 250 |
| XDU01000249 | lipoate--protein ligase A | *lplA* | 1005 | 334 |
| XDU01000257 | phosphopantetheine adenylyltransferase | *coaD* | 423 | 140 |
| XDU01000264 | tRNA sulfurtransferase ThiI | *thiI* | 1188 | 395 |
| XDU01000289 | thiamine pyrophosphokinase | - | 627 | 208 |
| XDU01000357 | FAD synthetase | - | 555 | 184 |
| XDU01000483 | lipoate--protein ligase A | *lplA* | 1038 | 345 |
| XDU01000501 | 5-formyltetrahydrofolate cyclo-ligase | - | 570 | 189 |
| XDU01000512 | methionine adenosyltransferase | *metK* | 1164 | 387 |
| XDU01000565 | nicotinate-nucleotide adenylyltransferase | - | 1104 | 367 |
| XDU01000567 | NAD(+) synthetase | *nadE* | 774 | 257 |
| XDU01000676 | nicotinate phosphoribosyltransferase | - | 1062 | 353 |
| XDU01000741 | bifunctional 5,10-methylene-tetrahydrofolate dehydrogenase/5,10-methylene-tetrahydrofolate cyclohydrolase | *mthfD* | 855 | 284 |
| XDU01000811 | dihydrofolate synthase | *folC* | 1110 | 369 |
| 27 | Nucleotide transport and metabolism |  |  |  |
| XDU01000006 | tRNA-specific adenosine deaminase | - | 444 | 147 |
| XDU01000009 | dTMP kinase | *tmk* | 642 | 213 |
| XDU01000088 | uracil phosphoribosyltransferase | *upp* | 624 | 207 |
| XDU01000111 | ribonucleoside-diphosphate reductase subunit beta | *nrdF* | 1020 | 339 |
| XDU01000112 | ribonucleotide reductase assembly protein NrdI | *nrdI* | 474 | 157 |
| XDU01000113 | ribonucleoside-diphosphate reductase subunit alpha | *nrdE* | 2163 | 720 |
| XDU01000114 | phosphoribosylpyrophosphate synthetase | *prs* | 1035 | 344 |
| XDU01000143 | CTP synthetase | *pyrG* | 1599 | 532 |
| XDU01000153 | thymidine kinase | *tdk* | 630 | 209 |
| XDU01000231 | guanylate kinase | *gmk* | 894 | 297 |
| XDU01000241 | hypoxanthine phosphoribosyltransferase | *hpt1* | 573 | 190 |
| XDU01000402 | non-canonical purine NTP pyrophosphatase | - | 600 | 199 |
| XDU01000413 | UMP kinase | *pyrH* | 714 | 237 |
| XDU01000435 | cytidine deaminase | - | 483 | 160 |
| XDU01000461 | uridine kinase | *udk* | 624 | 207 |
| XDU01000531 | adenine phosphoribosyltransferase | *apt* | 513 | 170 |
| XDU01000563 | deoxynucleoside kinase | - | 645 | 214 |
| XDU01000564 | 5'-methylthioadenosine nucleosidase | *pfs* | 657 | 218 |
| XDU01000595 | cytidylate kinase | *cmk* | 669 | 222 |
| XDU01000619 | deoxyguanosine kinase | - | 627 | 208 |
| XDU01000711 | adenylate kinase | *adk* | 642 | 213 |
| XDU01000792 | 2-deoxyribose-5-phosphate aldolase | *deoC* | 669 | 222 |
| XDU01000794 | pyrimidine-nucleoside phosphorylase | *deoA* | 1314 | 437 |
| XDU01000801 | hypoxanthine phosphoribosyltransferase | *hpt2* | 552 | 183 |
| XDU01000802 | adenylosuccinate lyase | *purB* | 1299 | 432 |
| XDU01000803 | adenylosuccinate synthase | *purA* | 1299 | 432 |
| XDU01000887 | purine-nucleoside phosphorylase | *deoD* | 654 | 217 |
| 30 | Inorganic ion transport and metabolism |  |  |  |
| XDU01000099 | magnesium-translocating P-type ATPase | *mgtA* | 2829 | 942 |
| XDU01000127 | ABC transporter permease | - | 1074 | 357 |
| XDU01000128 | peptide ABC transporter permease | - | 1011 | 336 |
| XDU01000129 | peptide ABC transporter ATP-binding protein | - | 1443 | 480 |
| XDU01000130 | ABC transporter ATP-binding protein | - | 1329 | 442 |
| XDU01000147 | membrane protein | - | 1494 | 497 |
| XDU01000172 | ABC transporter permease | - | 1245 | 414 |
| XDU01000173 | peptide ABC transporter permease | - | 1011 | 336 |
| XDU01000174 | ABC transporter ATP-binding protein | - | 1701 | 566 |
| XDU01000201 | chromate transporter | - | 621 | 206 |
| XDU01000202 | chromate transporter | - | 678 | 225 |
| XDU01000272 | ATPase | - | 2916 | 971 |
| XDU01000511 | copper homeostasis protein CutC | *cutC* | 684 | 227 |
| XDU01000517 | phosphate ABC transporter ATP-binding protein | *pstB* | 810 | 269 |
| XDU01000518 | phosphate ABC transporter permease | *pstC* | 2091 | 696 |
| XDU01000577 | CoA-disulfide reductase | - | 1787 | - |
| XDU01000682 | Fur family transcriptional regulator | - | 468 | 155 |
| XDU01000703 | energy-coupling factor transporter ATPase | *cbiO* | 909 | 302 |
| XDU01000704 | energy-coupling factor transporter ATPase | *cbiO* | 1227 | 408 |
| XDU01000742 | sodium transporter | - | 1602 | 533 |
| XDU01000743 | potassium transporter TrkA | *trkA* | 729 | 242 |
| XDU01000766 | ABC transporter permease | *phnE* | 1751 | - |
| XDU01000767 | ABC transporter ATP-binding protein | *phnC* | 750 | 249 |
| XDU01000796 | magnesium transporter | *mgtE* | 1404 | 467 |
| XDU01000848 | magnesium transporter | *mgtA* | 1116 | 371 |
| XDU01000849 | DNA-binding protein | *hup* | 934 | - |
| XDU01000850 | MFS transporter | - | 1428 | 475 |
| XDU01000861 | phosphonate ABC transporter substrate-binding protein | *phnD* | 1362 | 453 |
| XDU01000862 | phosphonate ABC transporter ATP-binding protein | *phnC* | 751 | - |
| XDU01000863 | phosphonate ABC transporter permease | *phnE* | 2732 | - |
| 34 | Amino acid transport and metabolism |  |  |  |
| XDU01000087 | serine hydroxymethyltransferase | *glyA* | 1242 | 413 |
| XDU01000100 | arginine deiminase | *arcA* | 1211 | - |
| XDU01000101 | ornithine carbamoyltransferase | *arcB* | 939 | 312 |
| XDU01000114 | phosphoribosylpyrophosphate synthetase | *prs* | 1035 | 344 |
| XDU01000126 | ABC transporter substrate-binding protein | - | 2955 | 984 |
| XDU01000127 | ABC transporter permease | - | 1074 | 357 |
| XDU01000128 | peptide ABC transporter permease | - | 1011 | 336 |
| XDU01000129 | peptide ABC transporter ATP-binding protein | - | 1443 | 480 |
| XDU01000130 | ABC transporter ATP-binding protein | - | 1329 | 442 |
| XDU01000135 | threonine dehydratase | *ilvA* | 1226 | - |
| XDU01000147 | membrane protein | - | 1494 | 497 |
| XDU01000167 | peptidase M17 | - | 1356 | 451 |
| XDU01000172 | ABC transporter permease | - | 1245 | 414 |
| XDU01000173 | peptide ABC transporter permease | - | 1011 | 336 |
| XDU01000174 | ABC transporter ATP-binding protein | - | 1701 | 566 |
| XDU01000175 | ABC transporter ATP-binding protein | - | 1878 | 625 |
| XDU01000197 | oligoendopeptidase F | - | 1794 | 597 |
| XDU01000199 | peptidase M17 | - | 1356 | 451 |
| XDU01000223 | spermidine/putrescine ABC transporter permease | *potC* | 3117 | 1038 |
| XDU01000224 | ABC transporter permease | *potB* | 993 | 330 |
| XDU01000225 | spermidine/putrescine import ATP-binding protein PotA | *potA* | 1056 | 351 |
| XDU01000371 | Xaa-Pro dipeptidase | - | 1077 | 358 |
| XDU01000454 | N-acetylneuraminate lyase | *nanA* | 888 | 295 |
| XDU01000455 | SSS family transporter | - | 1713 | 570 |
| XDU01000459 | peptidase M20 | - | 1350 | 449 |
| XDU01000503 | aminotransferase | - | 1239 | 412 |
| XDU01000687 | carbamate kinase | *arcC* | 932 | - |
| XDU01000688 | agmatine deiminase | *aguA* | 1096 | - |
| XDU01000689 | amino acid permease | - | 1404 | 467 |
| XDU01000690 | putrescine carbamoyltransferase | *argF* | 1098 | 365 |
| XDU01000770 | aminotransferase | - | 1195 | - |
| XDU01000845 | amino acid permease | - | 1578 | 525 |
| XDU01000850 | MFS transporter | - | 1248 | 415 |
| XDU01000853 | aspartate--ammonia ligase | *asnA* | 981 | 326 |
| 58 | Carbohydrate transport and metabolism |  |  |  |
| XDU01000028 | PTS lactose transporter subunit IIBt | - | 1550 | - |
| XDU01000032 | mannitol-1-phosphate 5-dehydrogenase | *mtlD* | 995 | - |
| XDU01000086 | ribose-5-phosphate isomerase | *rpiB* | 444 | 147 |
| XDU01000105 | PTS sugar transporter | *crr* | 2202 | 733 |
| XDU01000138 | glucosamine-6-phosphate deaminase | *agB2* | 768 | 255 |
| XDU01000139 | PTS sugar transporter subunit IIA | *nagE* | 1863 | 620 |
| XDU01000146 | fructose-1,6-bisphosphate aldolase, class II | *fba* | 894 | 297 |
| XDU01000147 | membrane protein | - | 1494 | 497 |
| XDU01000165 | PTS sugar transporter subunit IIC | *ptsG* | 1842 | 613 |
| XDU01000166 | alpha-xylosidase | - | 2268 | 755 |
| XDU01000185 | alpha-glycosidase | - | 1800 | 599 |
| XDU01000186 | cyclomaltodextrinase | - | 1794 | 597 |
| XDU01000191 | ABC transporter permease | - | 2435 | - |
| XDU01000192 | maltose ABC transporter permease | *malG* | 2549 | - |
| XDU01000193 | ABC transporter ATP-binding protein | - | 1086 | 361 |
| XDU01000194 | maltose phosphorylase | *mapA* | 2298 | 765 |
| XDU01000195 | glucan 1,6-alpha-glucosidase | - | 1605 | 534 |
| XDU01000238 | phosphopyruvate hydratase | - | 1356 | 451 |
| XDU01000242 | glycerol facilitator factor | *glpF* | 780 | 259 |
| XDU01000245 | 6-phosphofructokinase | *pfkA* | 981 | 326 |
| XDU01000246 | pyruvate kinase | *pyk* | 1438 | - |
| XDU01000258 | phosphoenolpyruvate--protein phosphotransferase | *ptsI* | 1722 | 573 |
| XDU01000259 | PTS glucose transporter subunit IIA | *crr* | 465 | 154 |
| XDU01000260 | dihydroxyacetone kinase subunit L | *dhaK2* | 627 | 208 |
| XDU01000287 | NAD(+) kinase | - | 798 | 265 |
| XDU01000290 | ribulose-phosphate 3-epimerase | *rpe* | 678 | 225 |
| XDU01000457 | sugar kinase | - | 876 | 291 |
| XDU01000458 | N-acetylmannosamine-6-phosphate 2-epimerase | *nanE* | 681 | 226 |
| XDU01000476 | N-acetylglucosamine-6-phosphate deacetylase | *nagA* | 1158 | 385 |
| XDU01000486 | ABC transporter permease | *gtsC* | 804 | 267 |
| XDU01000487 | glycerol ABC transporter permease | *gtsB* | 1005 | 334 |
| XDU01000488 | ABC transporter ATP-binding protein | *gtsA* | 1212 | 403 |
| XDU01000492 | PTS lactose transporter subunit IIB | *mtlA* | 1596 | 531 |
| XDU01000493 | PTS fructose transporter subunit IIA | - | 437 | - |
| XDU01000494 | 6-phosphogluconate dehydrogenase | - | 900 | 299 |
| XDU01000499 | glucose-6-phosphate isomerase | *pgi* | 1284 | 427 |
| XDU01000509 | mannose-6-phosphate isomerase | *pmi* | 930 | 309 |
| XDU01000608 | ribulose-phosphate 3-epimerase | *rpe* | 648 | 215 |
| XDU01000630 | transketolase | *tkt* | 1971 | 656 |
| XDU01000641 | fructose-bisphosphate aldolase | - | 846 | 281 |
| XDU01000642 | DeoR family transcriptional regulator | - | 735 | 244 |
| XDU01000643 | PTS sugar transporter subunit IIC | - | 1889 | - |
| XDU01000644 | phosphofructokinase | *fruK* | 942 | 313 |
| XDU01000665 | phosphoglycerate kinase | *pgk* | 1215 | 404 |
| XDU01000666 | type I glyceraldehyde-3-phosphate dehydrogenase | *gap* | 1017 | 338 |
| XDU01000733 | dihydroxyacetone kinase | *dhaK* | 1001 | - |
| XDU01000751 | phosphocarrier protein HPr | *ptsH* | 270 | 89 |
| XDU01000786 | glucosamine-6-phosphate deaminase | *nagB* | 729 | 242 |
| XDU01000787 | triose-phosphate isomerase | *tpiA* | 747 | 248 |
| XDU01000789 | 2,3-bisphosphoglycerate-independent phosphoglycerate mutase | *gpmI* | 1596 | 531 |
| XDU01000793 | phosphomannomutase | - | 1683 | 560 |
| XDU01000819 | PTS sugar transporter subunit IIB | - | 723 | 240 |
| XDU01000824 | PTS sugar transporter subunit IIB | - | 741 | 246 |
| XDU01000850 | MFS transporter | - | 1428 | 475 |
| XDU01000882 | PTS glucose transporter subunit IIBC | *ptsG* | 1742 | - |
| XDU01000890 | DeoR faimly transcriptional regulator | - | 701 | - |
| XDU01000891 | phosphofructokinase | - | 936 | 311 |
| XDU01000892 | PTS fructose transporter subunit IIABC | *fruB* | 2037 | 678 |
