# Supplementary material for: Genome-Wide Analysis of the First Sequenced Mycoplasma capricolum subsp. capripneumoniae Strain M1601
Source: G3 (Bethesda). 2017 Jul 27;7(9):2899–906. doi: 10.1534/g3.117.300085 (PMC5592918; doi:10.1534/g3.117.300085)
Supplement: Supplementary file 5 [file 2899TableS3.doc]

**Table S3 Transporter system of Mccp**

| Locus | Product | Gene | Gene length (bp) | Protein length (aa) |
| --- | --- | --- | --- | --- |
| XDU01000021 | ABC transporter ATP-binding protein | - | 1038 | 345 |
| XDU01000028 | PTS lactose transporter subunit IIB | *mtlA* | 1550 | - |
| XDU01000030 | PTS fructose transporter subunit IIA | - | 417 | 138 |
| XDU01000038 | sugar ABC transporter | - | 1608 | 535 |
| XDU01000039 | ABC transporter permease | - | 2532 | 843 |
| XDU01000040 | ABC transporter permease | - | 978 | 325 |
| XDU01000105 | PTS sugar transporter | *crr* | 2202 | 733 |
| XDU01000126 | ABC transporter substrate-binding protein | - | 2955 | 984 |
| XDU01000127 | ABC transporter permease | - | 1074 | 357 |
| XDU01000128 | peptide ABC transporter permease | - | 1011 | 336 |
| XDU01000129 | peptide ABC transporter ATP-binding protein | - | 1443 | 480 |
| XDU01000130 | ABC transporter ATP-binding protein | - | 1329 | 442 |
| XDU01000133 | ABC transporter ATP-binding protein | - | 1540 | - |
| XDU01000139 | PTS sugar transporter subunit IIA | *nagE* | 1863 | 620 |
| XDU01000165 | PTS sugar transporter subunit IIC | *ptsG* | 1842 | 613 |
| XDU01000172 | ABC transporter permease | - | 1245 | 414 |
| XDU01000173 | peptide ABC transporter permease | - | 1011 | 336 |
| XDU01000174 | ABC transporter ATP-binding protein | - | 1701 | 566 |
| XDU01000175 | ABC transporter ATP-binding protein | - | 1878 | 625 |
| XDU01000176 | ABC transporter substrate-binding protein | - | 3084 | 1027 |
| XDU01000191 | ABC transporter permease | - | 2435 | - |
| XDU01000192 | Maltose ABC transporter permease | *malG* | 2549 | - |
| XDU01000193 | ABC transporter ATP-binding protein | - | 1086 | 361 |
| XDU01000223 | spermidine/putrescine ABC transporter permease | *potC* | 3117 | 1038 |
| XDU01000224 | ABC transporter permease | *potB* | 993 | 330 |
| XDU01000225 | spermidine/putrescine import ATP-binding protein PotA | *potA* | 1056 | 351 |
| XDU01000258 | phosphoenolpyruvate--protein phosphotransferase | *ptsI* | 1722 | 573 |
| XDU01000259 | PTS glucose transporter subunit IIA | *crr* | 465 | 154 |
| XDU01000342 | ABC transporter permease | - | 5279 | - |
| XDU01000486 | ABC transporter permease | *gtsC* | 804 | 267 |
| XDU01000487 | glycerol ABC transporter permease | *gtsB* | 1005 | 334 |
| XDU01000488 | ABC transporter ATP-binding protein | *gtsA* | 1212 | 403 |
| XDU01000492 | PTS lactose transporter subunit IIB | *mtlA* | 1596 | 531 |
| XDU01000493 | PTS fructose transporter subunit IIA | - | 437 | - |
| XDU01000517 | phosphate ABC transporter ATP-binding protein | *pstB* | 810 | 269 |
| XDU01000518 | phosphate ABC transporter permease | *pstC* | 2091 | 696 |
| XDU01000519 | phosphate ABC transporter substrate-binding protein | - | 1212 | 403 |
| XDU01000543 | peptidase C39 | - | 1647 | 548 |
| XDU01000545 | ABC transporter permease | - | 5262 | 1753 |
| XDU01000573 | ABC transporter ATP-binding protein | - | 1854 | 617 |
| XDU01000574 | ABC transporter ATP-binding protein | - | 1872 | 623 |
| XDU01000606 | Na+ ABC transporter ATP-binding protein | - | 483 | 160 |
| XDU01000609 | PTS beta-glucoside transporter subunit | *sgaB* | 1809 | 602 |
| XDU01000610 | PTS fructose transporter subunit IIA | *sgaA* | 450 | 149 |
| XDU01000643 | PTS sugar transporter subunit IIC | *scrA* | 1888 | - |
| XDU01000691 | ABC transporter ATP-binding protein | - | 888 | 295 |
| XDU01000692 | ABC transporter permease | - | 765 | 254 |
| XDU01000700 | ABC transporter permease | - | 4146 | 1381 |
| XDU01000702 | ABC transporter permease | *cbiQ* | 1011 | 336 |
| XDU01000703 | energy-coupling factor transporter ATPase | *cbiO* | 909 | 302 |
| XDU01000704 | energy-coupling factor transporter ATPase | *cbiO* | 1227 | 408 |
| XDU01000751 | phosphocarrier protein HPr | - | 270 | 89 |
| XDU01000766 | ABC transporter permease | *phnE* | 1751 | - |
| XDU01000767 | ABC transporter ATP-binding protein | *phnC* | 750 | 249 |
| XDU01000768 | ABC transporter substrate-binding protein | *phnD* | 1467 | 488 |
| XDU01000819 | PTS sugar transporter subunit IIB | - | 723 | 240 |
| XDU01000824 | PTS sugar transporter subunit IIB | *malX* | 741 | 246 |
| XDU01000825 | hypothetical protein | *malX* | 618 | 205 |
| XDU01000833 | multidrug ABC transporter ATP-binding protein | - | 705 | 234 |
| XDU01000834 | ABC transporter permease | - | 1872 | 623 |
| XDU01000838 | ABC transporter permease | - | 1855 | - |
| XDU01000861 | phosphonate ABC transporter substrate-binding protein | *phnD* | 1362 | 453 |
| XDU01000862 | phosphonate ABC transporter ATP-binding protein | *phnC* | 751 | - |
| XDU01000863 | phosphonate ABC transporter permease | *phnE* | 2732 | - |
| XDU01000882 | PTS glucose transporter subunit IIBC | *ptsG* | 1742 | - |
| XDU01000892 | PTS fructose transporter subunit IIABC | *fruB* | 2037 | 678 |
