# Supplementary material for: Genome-Wide Analysis of the First Sequenced Mycoplasma capricolum subsp. capripneumoniae Strain M1601
Source: G3 (Bethesda). 2017 Jul 27;7(9):2899–906. doi: 10.1534/g3.117.300085 (PMC5592918; doi:10.1534/g3.117.300085)
Supplement: Supplementary file 6 [file 2899TableS4.doc]

**Table S4 Proteins involved in secretion system**

| Locus | Product | Gene | Gene length (bp) | Protein length (aa) |
| --- | --- | --- | --- | --- |
| XDU01000048 | preprotein translocase subunit SecA | *secA* | 2373 | 790 |
| XDU01000110 | preprotein translocase subunit SecG | *secG* | 285 | 94 |
| XDU01000123 | preprotein translocase subunit SecE | *secE* | 315 | 104 |
| XDU01000432 | peptidase A8 | *lspA* | 609 | 202 |
| XDU01000515 | signal recognition particle-docking protein FtsY | *ftsY* | 1275 | 424 |
| XDU01000532 | membrane protein | *secD* | 4215 | 1404 |
| XDU01000585 | signal recognition particle protein | *ffh* | 1344 | 447 |
| XDU01000712 | protein translocase subunit SecY | *secY* | 1449 | 482 |
| XDU01000909 | membrane protein insertase YidC | *yidC* | 1191 | 396 |
