# Supplementary material for: Genome-Wide Analysis of the First Sequenced Mycoplasma capricolum subsp. capripneumoniae Strain M1601
Source: G3 (Bethesda). 2017 Jul 27;7(9):2899–906. doi: 10.1534/g3.117.300085 (PMC5592918; doi:10.1534/g3.117.300085)
Supplement: Supplementary file 7 [file 2899TableS5.doc]

**Table S5 Predicted genes involved in DNA replication**

| Locus | Product | Gene | Gene length (bp) | Protein length (aa) |
| --- | --- | --- | --- | --- |
| XDU01000001 | ATPase involved in DNA replication initiation | *dnaA* | 1353 | 450 |
| XDU01000002 | DNA polymerase III subunit beta | *dnaN* | 1128 | 375 |
| XDU01000003 | ribonuclease M5 | - | 534 | 177 |
| XDU01000007 | DNA polymerase III, subunit gamma and tau | *dnaX* | 2010 | 669 |
| XDU01000008 | DNA repair protein RecM | *recM* | 591 | 196 |
| XDU01000010 | DNA polymerase III subunit delta | *holB* | 744 | 247 |
| XDU01000041 | DNA gyrase subunit A | *gyrA* | 2504 | - |
| XDU01000042 | DNA gyrase subunit B | *gyrB* | 1905 | 634 |
| XDU01000050 | 5'-3' exonuclease | - | 915 | 304 |
| XDU01000055 | Integrase | - | 919 | - |
| XDU01000069 | exodeoxyribonuclease VII large subunit | *xseA* | 1407 | 468 |
| XDU01000072 | deoxyribonuclease IV | *nfo* | 870 | 289 |
| XDU01000117 | replicative DNA helicase | *dnaB* | 1329 | 442 |
| XDU01000134 | hydrolase TatD | - | 797 | - |
| XDU01000230 | DNA methylase | - | 558 | 185 |
| XDU01000233 | DNA (cytosine-5-)-methyltransferase | *dcm* | 1026 | 341 |
| XDU01000240 | Holliday junction DNA helicase | - | 432 | 143 |
| XDU01000280 | excinuclease ABC subunit C | *uvrC* | 1755 | 584 |
| XDU01000292 | serine/threonine protein kinase | - | 1116 | 371 |
| XDU01000325 | DNA helicase | - | 1335 | 444 |
| XDU01000369 | PolC-type DNA polymerase III | *polC* | 4440 | 1479 |
| XDU01000385 | DNA methyltransferase | *dam* | 855 | 284 |
| XDU01000467 | Holliday junction DNA helicase RuvB | *ruvB* | 924 | 307 |
| XDU01000468 | Holliday junction DNA helicase RuvA | *ruvA* | 561 | 186 |
| XDU01000489 | exodeoxyribonuclease V subunit alpha | *recD* | 2196 | 731 |
| XDU01000490 | DNA topoisomerase IV subunit A | *parC* | 2700 | 899 |
| XDU01000491 | DNA topoisomerase IV subunit B | *parE* | 1932 | 643 |
| XDU01000508 | uracil-DNA glycosylase | *ung* | 657 | 218 |
| XDU01000534 | helicase | *deaD* | 1362 | 453 |
| XDU01000538 | DNA primase | *dnaG* | 1815 | 604 |
| XDU01000540 | DNA repair protein RecO | *recO* | 750 | 249 |
| XDU01000549 | ATPase AAA | *ycaJ* | 1239 | 412 |
| XDU01000578 | ribonuclease HII | *rnhB* | 624 | 207 |
| XDU01000587 | recombinase RecA | *recA* | 1037 | - |
| XDU01000592 | DNA-binding protein | *hupB* | 273 | 90 |
| XDU01000636 | exodeoxyribonuclease V subunit alpha | *recD* | 954 | 317 |
| XDU01000648 | Putative helicase | - | 477 | 158 |
| XDU01000667 | primosomal protein | *dnaI* | 936 | 311 |
| XDU01000668 | chromosome replication initiation/membrane attachment protein | *dnaB* | 1185 | 394 |
| XDU01000669 | DNA-formamidopyrimidine glycosylase | *mutM* | 825 | 274 |
| XDU01000670 | DNA polymerase I | *polA* | 2736 | 911 |
| XDU01000671 | DNA polymerase III subunit alpha | *dnaE* | 2961 | 986 |
| XDU01000747 | DNA ligase (NAD(+)) LigA | *ligA* | 2007 | 668 |
| XDU01000752 | ATPase AAA | *pcrA* | 2169 | 722 |
| XDU01000804 | TatD family hydrolase | *tatD* | 801 | 266 |
| XDU01000808 | DNA polymerase III subunit delta | *holA* | 951 | 316 |
| XDU01000809 | excinuclease ABC subunit B | *uvrB* | 1998 | 665 |
| XDU01000810 | excinuclease ABC subunit A | *uvrA* | 2841 | 946 |
| XDU01000829 | DNA topoisomerase I | *topA* | 1932 | 643 |
| XDU01000849 | DNA-binding protein | *hupB* | 934 | - |
