# Supplementary material for: Genome-Wide Analysis of the First Sequenced Mycoplasma capricolum subsp. capripneumoniae Strain M1601
Source: G3 (Bethesda). 2017 Jul 27;7(9):2899–906. doi: 10.1534/g3.117.300085 (PMC5592918; doi:10.1534/g3.117.300085)
Supplement: Supplementary file 8 [file 2899TableS6.doc]

**Table S6 Predicted genes involved in transcription**

| Locus | Product | Gene | Gene length (bp) | Protein length (aa) |
| --- | --- | --- | --- | --- |
| XDU01000012 | RpiR family transcriptional regulator | - | 822 | 273 |
| XDU01000027 | ATPase | - | 1389 | 462 |
| XDU01000031 | RpiR family transcriptional regulato | *glvR* | 798 | 265 |
| XDU01000070 | transcription antitermination factor NusB | *nusB* | 399 | 132 |
| XDU01000082 | DNA-directed RNA polymerase subunit beta | *rpoB* | 3864 | 1287 |
| XDU01000083 | DNA-directed RNA polymerase subunit beta | *rpoC* | 3768 | 1255 |
| XDU01000103 | ROK family transcriptional regulator | *glk* | 963 | 320 |
| XDU01000109 | ribonuclease R | - | 2115 | 704 |
| XDU01000124 | transcription antitermination protein NusG | *nusG* | 651 | 216 |
| XDU01000196 | GntR family transcriptional regulator | *gntR* | 721 | - |
| XDU01000279 | transcription elongation factor GreA | *greA* | 474 | 157 |
| XDU01000292 | serine/threonine protein kinase | - | 1116 | 371 |
| XDU01000365 | RNA-binding protein | - | 282 | 93 |
| XDU01000366 | transcription termination/antitermination protein NusA | *nusA* | 1677 | 558 |
| XDU01000406 | HrcA family transcriptional regulator | *hrcA* | 1023 | 340 |
| XDU01000457 | sugar kinase | *nanK* | 876 | 291 |
| XDU01000482 | deacetylase SIR2 | - | 852 | 283 |
| XDU01000526 | ribonuclease 3 | *rnc* | 699 | 232 |
| XDU01000530 | guanosine-3',5'-bis(diphosphate) 3'-pyrophosphohydrolase | *relA* | 2244 | 747 |
| XDU01000534 | helicase | *deaD* | 1362 | 453 |
| XDU01000537 | RNA polymerase sigma factor RpoD | *rpoD* | 1521 | 506 |
| XDU01000621 | SMC-Scp complex subunit ScpB | *scpB* | 630 | 209 |
| XDU01000642 | DeoR family transcriptional regulator | *fruR* | 735 | 244 |
| XDU01000655 | hypothetical protein | - | 1413 | 470 |
| XDU01000682 | Fur family transcriptional regulator | *fur* | 468 | 155 |
| XDU01000706 | DNA-directed RNA polymerase subunit alpha | *rpoA* | 954 | 317 |
| XDU01000890 | DeoR faimly transcriptional regulator | *fruR* | 701 | - |
