# Supplementary material for: Genome-Wide Analysis of the First Sequenced Mycoplasma capricolum subsp. capripneumoniae Strain M1601
Source: G3 (Bethesda). 2017 Jul 27;7(9):2899–906. doi: 10.1534/g3.117.300085 (PMC5592918; doi:10.1534/g3.117.300085)
Supplement: Supplementary file 9 [file 2899TableS7.doc]

**Table S7 The genes predicted to be participated in translation**

| Locus | Product | Gene | Gene length (bp) | Protein length (aa) |
| --- | --- | --- | --- | --- |
| XDU01000004 | 16S rRNA | *rsmA* | 801 | 266 |
| XDU01000006 | tRNA-specific adenosine deaminase | - | 444 | 147 |
| XDU01000024 | 30S ribosomal protein S6 | *rpsF* | 414 | 137 |
| XDU01000026 | 30S ribosomal protein S18 | *rpsR* | 228 | 75 |
| XDU01000036 | methionine--tRNA ligase | *metG* | 1530 | 509 |
| XDU01000068 | tRNA synthetase RNA-binding protein | - | 811 | - |
| XDU01000077 | 50S ribosomal protein L11 | *rplK* | 429 | 142 |
| XDU01000078 | 50S ribosomal protein L1 | *rplA* | 681 | 226 |
| XDU01000079 | 50S ribosomal protein L10 | *rplJ* | 498 | 165 |
| XDU01000080 | 50S ribosomal protein L7/L12 | *rplL* | 369 | 122 |
| XDU01000115 | aminoacyl-tRNA hydrolase | *pth* | 561 | 186 |
| XDU01000116 | 50S ribosomal protein L9 | *rplI* | 444 | 147 |
| XDU01000120 | cysteine--tRNA ligase | *cysS* | 1326 | 441 |
| XDU01000121 | 23S rRNA (guanosine(2251)-2'-O)-methyltransferase RlmB | - | 735 | 244 |
| XDU01000122 | 50S ribosomal protein L33 | *rpmG* | 162 | 53 |
| XDU01000140 | glutamate--tRNA ligase | *gltX* | 1452 | 483 |
| XDU01000150 | 50S ribosomal protein L31 | *rpmE* | 282 | 93 |
| XDU01000154 | peptide chain release factor 1 | *prfA* | 1092 | 363 |
| XDU01000155 | N5-glutamine S-adenosyl-L-methionine-dependent methyltransferase | *hemK* | 849 | 282 |
| XDU01000157 | translation factor | *sua5* | 501 | 166 |
| XDU01000161 | 30S ribosomal protein S12 | *rpsL* | 420 | 139 |
| XDU01000162 | 30S ribosomal protein S7 | *rpsG* | 468 | 155 |
| XDU01000163 | elongation factor G | *fusA* | 2070 | 689 |
| XDU01000164 | elongation factor Tu | *tufA* | 1188 | 395 |
| XDU01000169 | alanine--tRNA ligase | *alaS* | 2691 | 896 |
| XDU01000226 | 50S ribosomal protein L20 | *rplT* | 360 | 119 |
| XDU01000227 | 50S ribosomal protein L35 | *rpmI* | 192 | 63 |
| XDU01000228 | translation initiation factor IF-3 | *infC* | 546 | 181 |
| XDU01000229 | peptide deformylase | *def* | 603 | 200 |
| XDU01000232 | Sun family protein | *sun* | 1270 | - |
| XDU01000247 | threonine--tRNA ligase | *thrS* | 1920 | 639 |
| XDU01000262 | 30S ribosomal protein S4 | *rpsD* | 627 | 208 |
| XDU01000288 | valine--tRNA ligase | *valS* | 2619 | 872 |
| XDU01000348 | proline--tRNA ligase | *proS* | 1425 | 474 |
| XDU01000353 | aspartate--tRNA ligase | *aspS* | 1728 | 575 |
| XDU01000354 | histidine--tRNA ligase | *hisS* | 1245 | 414 |
| XDU01000355 | ribosome-binding factor A | *rbfA* | 354 | 117 |
| XDU01000356 | pseudouridine synthase | *truB* | 877 | - |
| XDU01000360 | 30S ribosomal protein S15 | *rpsO* | 267 | 88 |
| XDU01000363 | translation initiation factor IF-2 | *infB* | 1863 | 620 |
| XDU01000364 | 50S ribosomal protein L7ae | *rpl7ae* | 300 | 99 |
| XDU01000372 | tryptophan--tRNA ligase | *trpS* | 1011 | 336 |
| XDU01000403 | RNA methyltransferase | - | 546 | 181 |
| XDU01000410 | 30S ribosomal protein S2 | *rpsB* | 876 | 291 |
| XDU01000411 | elongation factor Ts | *tsf* | 888 | 295 |
| XDU01000414 | ribosome recycling factor | *frr* | 549 | 182 |
| XDU01000415 | arginine--tRNA ligase | *argS* | 1665 | 554 |
| XDU01000421 | phenylalanine--tRNA ligase subunit alpha | *pheS* | 1053 | 350 |
| XDU01000422 | phenylalanine--tRNA ligase subunit beta | *pheT* | 2385 | 794 |
| XDU01000424 | 50S ribosomal protein L32 | *rpmF* | 180 | 59 |
| XDU01000431 | isoleucine--tRNA ligase | *ileS* | 2727 | 908 |
| XDU01000451 | 50S ribosomal protein L21 | *rplU* | 303 | 100 |
| XDU01000453 | 50S ribosomal protein L27 | *rpmA* | 282 | 93 |
| XDU01000496 | rRNA methyltransferase | - | 768 | 255 |
| XDU01000510 | methylenetetrahydrofolate--tRNA-(uracil(54)- C(5))-methyltransferase (FADH(2)-oxidizing) TrmFO | *trmFO* | 1317 | 438 |
| XDU01000522 | 50S ribosomal protein L28 | *rpmB* | 198 | 65 |
| XDU01000534 | helicase | *helicase* | 1362 | 453 |
| XDU01000539 | glycine--tRNA ligase | *glyS* | 1371 | 456 |
| XDU01000553 | elongation factor P | *efp* | 555 | 184 |
| XDU01000554 | methionyl-tRNA formyltransferase | *fmt* | 954 | 317 |
| XDU01000557 | tRNA 2-thiouridine(34) synthase MnmA | *trmU* | 1128 | 375 |
| XDU01000580 | 50S ribosomal protein L19 | *rplS* | 393 | 130 |
| XDU01000581 | tRNA (guanosine(37)-N1)-methyltransferase TrmD | *trmD* | 723 | 240 |
| XDU01000583 | 30S ribosomal protein S16 | *rspP* | 327 | 108 |
| XDU01000620 | ribosomal large subunit pseudouridine synthase B | *rluB* | 759 | 252 |
| XDU01000632 | methylenetetrahydrofolate--tRNA-(uracil-5-)-methyltransferase | *trmFO2* | 1272 | 423 |
| XDU01000675 | tyrosine--tRNA ligase | *tyrS* | 1245 | 414 |
| XDU01000695 | leucine--tRNA ligase | *leuS* | 2415 | 804 |
| XDU01000698 | 30S ribosomal protein S9 | *rpsI* | 399 | 132 |
| XDU01000699 | 50S ribosomal protein L13 | *rplM* | 456 | 151 |
| XDU01000701 | tRNA pseudouridine(38,39,40) synthase TruA | *truA* | 750 | 249 |
| XDU01000705 | 50S ribosomal protein L17 | *rplQ* | 360 | 119 |
| XDU01000707 | 30S ribosomal protein S11 | *rpsK* | 390 | 129 |
| XDU01000708 | 30S ribosomal protein S13 | *rpsM* | 366 | 121 |
| XDU01000709 | translation initiation factor IF-1 | *infA* | 225 | 74 |
| XDU01000710 | methionine aminopeptidase | *map* | 756 | 251 |
| XDU01000713 | 50S ribosomal protein L15 | *rplO* | 438 | 145 |
| XDU01000714 | 30S ribosomal protein S5 | *rpsE* | 765 | 254 |
| XDU01000715 | 50S ribosomal protein L18 | *rplR* | 351 | 116 |
| XDU01000716 | 50S ribosomal protein L6 | *rplF* | 543 | 180 |
| XDU01000717 | 30S ribosomal protein S8 | *rpsH* | 390 | 129 |
| XDU01000718 | 30S ribosomal protein S14 | *rpsN* | 186 | 61 |
| XDU01000719 | 50S ribosomal protein L5 | *rplE* | 543 | 180 |
| XDU01000720 | 50S ribosomal protein L24 | *rplX* | 327 | 108 |
| XDU01000721 | 50S ribosomal protein L14 | *rplN* | 369 | 122 |
| XDU01000722 | 30S ribosomal protein S17 | *rpsQ* | 258 | 85 |
| XDU01000723 | 50S ribosomal protein L29 | *rpmC* | 417 | 138 |
| XDU01000724 | 50S ribosomal protein L16 | *rplP* | 414 | 137 |
| XDU01000725 | 30S ribosomal protein S3 | *rpsC* | 702 | 233 |
| XDU01000726 | 50S ribosomal protein L22 | *rplV* | 336 | 111 |
| XDU01000727 | 30S ribosomal protein S19 | *rspS* | 267 | 88 |
| XDU01000728 | 50S ribosomal protein L2 | *rplB* | 846 | 281 |
| XDU01000729 | 50S ribosomal protein L23 | *rplW* | 285 | 94 |
| XDU01000730 | 50S ribosomal protein L4 | *rplD* | 627 | 208 |
| XDU01000731 | 50S ribosomal protein L3 | *rplC* | 672 | 223 |
| XDU01000732 | 30S ribosomal protein S10 | *rpsJ* | 309 | 102 |
| XDU01000744 | aspartyl/glutamyl-tRNA amidotransferase subunit B | *gatB* | 1440 | 479 |
| XDU01000745 | aspartyl/glutamyl-tRNA amidotransferase subunit A | *gatA* | 1458 | 485 |
| XDU01000749 | RNA pseudouridine synthase | *rluC* | 909 | 302 |
| XDU01000771 | reactive intermediate/imine deaminas | - | 402 | 133 |
| XDU01000841 | GTP-binding protein YchF | *ychF* | 1095 | 364 |
| XDU01000854 | 30S ribosomal protein S20 | *rpsT* | 246 | 81 |
| XDU01000860 | asparagine--tRNA ligase | *asnS* | 1365 | 454 |
| XDU01000872 | lysine--tRNA ligase | *lysS* | 1503 | 500 |
| XDU01000873 | tRNA-dihydrouridine synthase | *dus* | 975 | 324 |
| XDU01000875 | serine--tRNA ligase | *serS* | 1269 | 422 |
| XDU01000910 | ribonuclease P protein component | *rnpA* | 330 | 109 |
| XDU01000911 | 50S ribosomal protein L34 | *rpmH* | 135 | 44 |
