# Supplementary material for: Genome-Wide Analysis of the First Sequenced Mycoplasma capricolum subsp. capripneumoniae Strain M1601
Source: G3 (Bethesda). 2017 Jul 27;7(9):2899–906. doi: 10.1534/g3.117.300085 (PMC5592918; doi:10.1534/g3.117.300085)
Supplement: Supplementary file 11 [file 2899TableS9.doc]

**Table S9 The genome information of Mccp strain M1601 and other four partial annotated Mccp strains**

| Strains | Accession No.^a^ | Genome size (bp)^a^ | Genes^a^ | CDS^a^ | Pseudo Genes*^a^* | rRNAs*^a^* | tRNAs*^a^* | ncRNA*^a^* |
| --- | --- | --- | --- | --- | --- | --- | --- | --- |
| M1601 | NZ_CP017125 | 1,016,707 | 915 | 713 | 163 | 6 | 30 | 3 |
| F38 | NZ_LN515398 | 1,016,760 | 914 | 729 | 146 | 6 | 30 | 3 |
| ILRI181 | NZ_LN515399 | 1,017,183 | 911 | 714 | 161 | 6 | 30 | 0 |
| 87001 | NZ_CP006959 | 1,017,333 | 908 | 700 | 172 | 6 | 30 | 0 |
| 9231 | NZ_LM995445 | 1,017,293 | 914 | 728 | 150 | 6 | 30 | 0 |

*^a^* Data from NCBI databases on Jan 10, 2017.
